# Mercury and Other Remedies in the Treatment of Tuberculous Meningitis

**Published:** 1906-03-31

**Authors:** 


[1] Lancet, March 24, 1906.

### MERCURY AND OTHER REMEDIES IN THE TREATMENT OF TUBERCULOUS MENINGITIS.

Dr. W. H. Whitcombe-Brown [1] records a case in which, after a diagnosis of tuberculous meningitis, sustained by the opinion of three other practitioners, the patient made a good recovery under the free administration of mercury. Of course, it must be allowed that in these circumstances the diagnosis may be open to question, but at the same time the facts of the case appear to exclude the possibility of any other interpretation. The patient was a child of twelve months, who, after a period of febrile disturbance, accompanied by vomiting, gradually became comatose, with loss of light response in the pupils and general convulsive seizures. Lumbar puncture was not permitted. Mercurial inunction was commenced comparatively early in the course of the disease, a piece of the ointment the size of a filbert-nut being rubbed in twice a day for a fortnight, and afterwards once a day.

At the same time a mixture containing perchloride of mercury with iodide and bromide of potassium was given by the mouth. Every effort was made to maintain the nutrition of the child, and under this treatment consciousness gradually returned, and the patient ultimately made a complete recovery. There is, of course, no *a priori* reason why the development of tubercle in the cerebral meninges should not be checked and the symptoms dependent on it brought to a happy conclusion. Indeed, in view of what is known of tubercle in the lungs, peritoneum, and elsewhere, the presumption is in an exactly opposite direction. The gloomy prognosis which attends tuberculous meningitis is, however, the result of an experience which cannot be questioned. Nevertheless, such cases as the above do afford a certain qualification to the general verdict, and they ought to encourage more energy and resolution in treatment. Probably were mercurial inunction commenced at an earlier date and pursued with more thoroughness the results would be less unsatisfactory.

It must be remembered that the possibility of recovery from tuberculous meningitis does not depend merely on clinical records such as the above, which is, by the way, far from being an isolated case. Evidence has been present in other instances in the shape of tubercles in the choroid, and in some exceptional cases post-mortem examination has shown that at some long antecedent date the subject had had an attack of basal meningitis. Obviously such records add to the probability of the correctness of the diagnosis in occasional cases where the clinical conditions suggest a tuberculous meningitis and the patient makes a good recovery.

Another occasional feature in the natural history of the disease also points in the same direction. This is the occurrence of a remission of the symptoms, not limited to a week or so, but extending over many months. No doubt such an experience is rare, but, even admitting this, it occurs with sufficient frequency to show that the disease is not absolutely and invariably free from all tendency to recovery, and this again re-inforces the suggestion that it is a mistake to allow a hopeless outlook to paralyse all attempts at serious treatment.

Among remedies other than mercury which have been advised in the treatment of tuberculous meningitis may be mentioned ergot and iodoform. Dr. James Gilroy [2] has recorded some cases in which the diagnosis seemed fairly well assured and in which recovery followed the internal administration of liquid extract of ergot with potassium bromide and iodide and the application of an iodoform ointment to the shaved scalp. Dr. Gilroy places principal stress on the ergot, and though it is difficult to reconcile this with modern pharmacological views, it must be remembered that in questions of therapeutics the last word is spoken at the bedside and not in the laboratory. As an example of the possible value of iodoform ointment in tuberculous meningitis reference may be made to a case quoted by Dr. Burney Yeo.[3] The patient was a boy of eight years; four of his brothers and sisters had died with symptoms of the disease, and on his mother's side there was a history of phthisis pulmonalis. The lad suffered from headache, torpor, convulsions, strabismus, and pyrexia. As he was steadily getting worse, the head was shaved and an ointment of iodoform (1 in 10) was thoroughly rubbed in, the head afterwards being covered with an oil-skin cap. This was repeated three or four times a day, and on this feature in the scheme of treatment considerable stress is placed. The boy gradually improved, consciousness returned, and in the end he made a good recovery. There were evidences of irritation from absorption of iodoform, such as coryza, redness of the eyelids, and irritable cough; but these disappeared when the ointment was discontinued.

[1] Lancet, March 24, 1906. [2] Ibid., Jan. 30, 1904. [3] Ibid., March 16, 1901.

